# Infective Diseases

**Published:** 1895-03-09

**Authors:** 


### Progress in Medicine.

### INFECTIVE DISEASES.

**Influenza.**—No material advance in our knowledge has been made since the papers of Grainger Stewart, Samuel West, and Eade, but much discussion of the sequelæ and complications has taken place. Pfeiffer's bacillus has been frequently isolated. It is rare in the blood, and, when found, appears to be in a weak or dying state.[1] Pïelicke found that the large pseudo-bacillus under cultivation developed into the typical form.[2] In typical pneumonia, Fraenkel's pneumococcus was found; in atypical ones, the influenza bacillus seems to favour the growth of strepto- and stapylo-cocci. Leyden has[3] always found Fraenkel's coccus in the lungs of those dead of influenza, but Fraenkel denies their presence, though he insists that the sputa are rusty. Klein[4] found that inoculation of animals gave practically negative results. The bacilli only grow at high temperatures, and die off in about twenty-four hours, so that their dispersion in dust is unexplained.[5] Mossé found recently that previous injections of quinine made rabbits resistant to injections of influenza cultures,[6] while others unprotected were attacked after his injections. Teissier insists on the polymorphous character of the bacilli,[7] and finds them most easily in the urine during defervescence of the complaint. He succeeded with rabbits in producing the disease by injection, and in cultivating the microbe in sterilized water for months. However, his results are so opposed to those of others that some caution is felt in accepting them, though Charrin[8] and Roux apparently agree with him. It is probable that the rabbits were affected only by the toxins, as Klein remarks that during the great epidemic there was no disease noticed in the zoological gardens or elsewhere in animals. G. Stewart believes that the bacilli lying for the most part outside the tissues, as in diphtheria, favour the growth of other organisms,[9] and excrete certain poisons which cause the nerve lesions. These ferments or poisons pass through one stage of elaboration in the spleen. The moist sputa probably retains their virulence for some time.

The most remarkable clinical point is, perhaps, the prolonged sub-normal temperature after the febrile stage, with a slow pulse and respiration.[10] This slowness in both sometimes occurs in periodic paroxysms, even during pneumonia, and is accompanied by much collapse. The pneumonia temperature may be low and remittent, and the condition is often marked by cyanosis, sweating, and extreme prostration. W. A. Wills, the registrar of Middlesex Hospital, however, remarks that lobar pneumonia following influenza[11] is certainly not always atypical, though probably rather more cases end by lysis than in pure pneumonia. Mortimer Granville goes[12] so far on the other hand as to regard a lobular pneumonia as the essential lesion of influenza, which is universally present. Burgess inclines to the view that the form of pneumonia is peculiar to the disease.[13] He had noticed in one case consolidation of a lower lobe without breath-sounds, vocal resonance, or the presence of fluid, and in another a very high tympanic note over the upper lobe, both of which are rare; but, besides this, he holds that the disease runs a specific course. Eade regards the affection as a bronchitis. The rashes include erythema, vesicles on the palate, urticaria and herpes. The albuminuria[14] frequently following it usually passes away without harm. The cardiac complications have been discussed by Sansom, who notices: (1) anginal attacks; (2) tachycardia; (3) brachycardia; (4) irregularity; and (5) possibly organic lesions. The anginal attacks, though severe, have not the associated signs of true angina,[15] and may probably be due to a neuritis of the cardiac plexuses. The other forms arise chiefly from a neuritis of the roots of the vagus with possibly other lesions. Together with irregularity there may be present disturbances of hearing, outbreaks of gout and acute dyspepsia, and even the symptoms of Graves' disease. Treatment is unsatisfactory, the ordinary cardiac tonics are quite useless, but the continuous current from the back to the front of the neck is of some value. No microscopic appearances were mentioned by the author. In support of this theory of neuritis, Macphall[16] mentions the case of a man, suffering from chronic mania, who developed typical peripheral neuritis after a second attack of influenza which improved under the usual treatment, and

Stembo gives instances of bulbar paralysis and motor aphasia[17] following similar attacks. Sansom elsewhere quotes cases where pathological changes were actually found post-mortem in the cord, and details a spinal case of his own which commenced with influenza of a so-called arthritic type.[18] There was paresis of the arms with some inco-ordination, great weakness of the legs with exaggerated deep reflexes and ankle clonus. Two fatal instances of violent pain in the head after influenza, in one of which retinal hæmorrhages were observed, are noted by Mortimer Rowland.[19] There was obstinate constipation, later on a general improvement; afterwards, the pain came on suddenly, with fainting, but without vomiting. The precise way in which the poison effects these various results is, however, very obscure. Thrombosis, both in veins and arteries, is fairly common; and Althaus[20] refers to thirty-four instances of it, which he would explain as caused by the breaking up of the numerous white corpuscles. In connection with these blood changes both Stewart and Sansom note[21] that purpura hæmorrhagica may occur as a sequela, and hæmaturia appeared in an Egyptian epidemic. The neural changes are probably due to a direct action of the poison on the nerves themselves.

**Microbial Poisons.**—It has been recently shown in Bouchard's laboratory that in animals poisoned by lead, alcohol, tuberculin, and other toxic substances, multiplication of the germs of an infective disorder is more rapid than usual, hence the rapidity with which debilitated persons succumb.[22] There is little or no destruction of the germs at the point of inoculation as in healthy persons. But, again, if any organ be injured and bacteria be injected into the circulation they are found in excess in the damaged tissues, or if lead be administered to animals with joint or lung lesions, the lead is found by the ordinary chemical tests to be deposited in the diseased spot. A similar deposit occurs in muscles of which the nerve supply is destroyed, hence in some way (not by the action of leucocytosis, however) injured tissues are as it were compelled to absorb any poisons in the circulation. Similar clinical facts in gout and other diseases are well-known.

The chief microbial poisons at present known are albumoses, peptones, nucleo-albumens, and alkaloids. In most cases the bacteria give off a ferment which at a distance from its source reacts upon the tissues of the host so as to split off various toxic proteids. In some forms, as perhaps in cholera and typhoid, there remains only an alkaloid; in others, as in diphtheria, we have a powerful albumose. Normally poisonous peptones are formed in the process of digestion, but their entrance into the blood is prevented by a special mechanism. Recent researches have shown that poisonous proteids akin to those produced by bacteria are formed both by higher vegetables, such as the seeds of the jequirity plant, and by spiders and snakes. Halliburton points out[23] that the poison elaborated by the glands of some snakes is neither a ferment, an alkaloid, an acid, nor an organism, but consists of two albumoses. A grain of these injected into the vessels causes the death of an ordinary rabbit in about 100 seconds, but there is reason to think that the action is indirect, and that the albumoses acting on the endothelium set free a nucleo-albumen which produces instantaneous clotting. Calmette has claimed that serum obtained[24] as in diphtheria can be used as a cure, or to produce immunity. Indeed, the injection of 30 c.c. of a solution of 1 in 12 hypochlorite of calcium quite freshly prepared is an antidote to the cobra or viper bite. As from 21,000 to 26,000 of our Indian fellow-subjects are killed by snakes every year the subject is one of deep interest in itself and not only as a development of serum therapeutics. The poison gradually loses its virulence[25] by drying, or by boiling for a short time. The blood of vipers contains a toxic principle akin to the venom, but Phisalix and Bertrand show that the poison glands do not merely eliminate a substance contained in the blood, but are the true source of it.[26] The blood is through the absorbed venom rendered immune should the snake inflict any accidental scratch upon itself. Further investigations are being carried out at the Institute Pasteur. The action of two microbes in combination is another subject which is now attracting attention. Thus Mosny and Marcano find that rabbits who have survived a small dose of the toxins of the staphylococcus pyogenes, perish later on through the migration and virulent action of the bacteria coli which were normally present in their intestines.[27] There were none of the ordinary staphylococcal lesions, but the bacteria coli became capable of causing peritonitis and diarrhœa. Fortunately, the bacteria of most of our infective diseases have apparently no specially favouring action on one another. The clinical results of the concurrence of two fevers in the same patient have been carefully examined by Caiger and others. Two, three, and even four diseases were observed concurrently.[28] There appeared to be no antagonism, but they only afforded increased susceptibility either by depressing the general condition, or by facilitating the development of another disorder by local inflammation, such as that of the tonsils, or of the bronchi. Thus diphtheria was specially serious after the bronchial inflammation of measles. In the case of the bacterium coli and the erysipelas germ, there was much evidence of intensified virulence in the presence of certain other organisms. Thus to the bacterium coli in the presence of typhoid toxin the secondary lesions of typhoid fever are due.

[1] Lancet, Mar. 31. [2] B.M.J., July 7. [3] Med. Week, July 1. [4] Med. Chron., May. [5] Am. M.S. Bullet., Aug. 15. [6] Lanc., Nov. 3. [7] Med. Week, Sept. 28. [8] Med. Week, Ap. 21. [9] B.M.J., Aug. 4. [10] Lanc., Ap. 28. [11] Lanc., May 12. [12] A.J. Med. Sc., Dec. [13] Lanc., June 2. [14] B.M.J., Ap. 21. [15] Med. Week, June 15 and Aug. 10. [16] Practit., June. [17] A.J. Med. Sc., Oct. [18] Lanc., Sept. 8. [19] Lanc., July 21. [20] Lanc., June 9. [21] Lanc., June 2. [22] Med. Week, Aug. 17-24. [23] Sc. Progress, Sept. [24] Merc. Med., No. 13, '94. [25] Med. Chron., Aug. [26] Med. Week, Dec. 7. [27] Med. Week, Dec. 3. [28] B.M.J., May 5.

